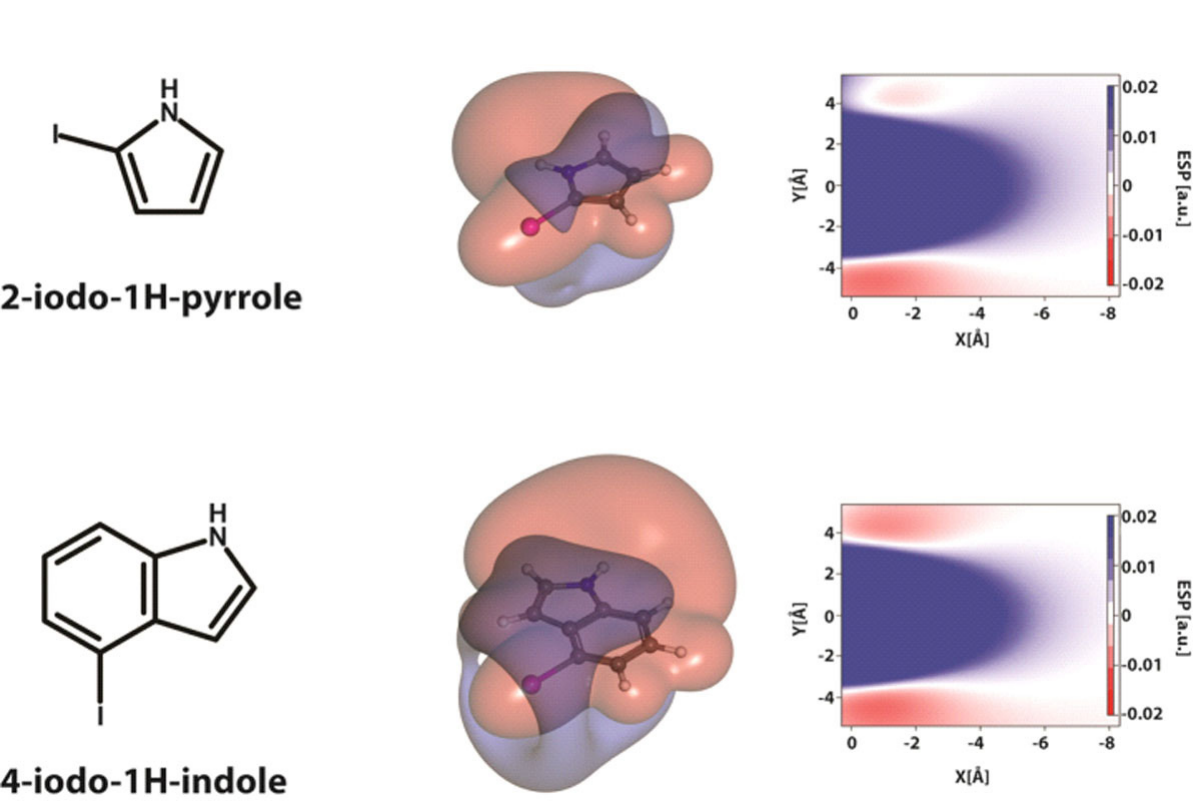# Scaffold dependencies for halogen bonding: quantum mechanical investigation of nitrogen-bearing heterocycles

**DOI:** 10.1186/1758-2946-6-S1-P16

**Published:** 2014-03-11

**Authors:** A Lange, MO Zimmermann, MF Boeckler

**Affiliations:** 1 Laboratory for Mol. Design& Pharm.Biophysics, Eberhard Karls University Tuebingen, Auf der Morgenstelle 8, 72076 Tuebingen, Germany

## 

Halogen bonding is a rather new but promising type of interaction for the drug discovery process. It is rather directional and involves an electron donor as binding partner. Employing quantum chemical calculations [1-3], we explore the applicability of halogen bonds in molecular design with respect to halogen-enriched fragment libraries (HEFlibs) [4]. Computational studies on protein-ligand complexes have been reported indicating varying halogen-bond strengths that depend on the chemical nature of the ligand and the surrounding binding pocket. The strength and behavior of a halogen bond certainly depends on the core scaffold to which the halogen is attached. Due to the relative novelty of halogen bonding in medicinal chemistry, only a few experimental studies exist [4-6]. Therefore, we computed 30 different nitrogen-bearing heterocycles substituted by chlorine, bromine, or iodine (in total 459 structures) to increase our understanding of scaffold-based tuning of halogen bonds (see Figure 1 below for a few examples). For each structure the electrostatic-potential isosurfaces will be plotted (3D and 2D) to illustrate changes in halogen bond strength and the appearance of binding motifs in certain substituted heterocycles. Furthermore, we aim at providing simplified rules and models to estimate halogen bonding strength based on the molecular electrostatic potentials. Our data highlights that the use of an appropriate scaffold exerts a most important influence on the applicability of a halogen bonding.

**Figure 1:**